# Catalytic Performance of Oxydianiline-Derived Polybenzoxazine in the Cycloaddition of CO_2_ with Epoxides for Selective and Cleaner Production of Cyclic Carbonates

**DOI:** 10.3390/ijms26031111

**Published:** 2025-01-27

**Authors:** Rafik Rajjak Shaikh, Mohammad Yazdanpanah, Isak Rajjak Shaikh, Rais Ahmad Khan, Supareak Praserthdam, Piyasan Praserthdam

**Affiliations:** 1 Center of Excellence on Catalysis and Catalytic Reaction Engineering (CECC), Department of Chemical Engineering, Faculty of Engineering, Chulalongkorn University, Bangkok 10330, Thailand; rafik.rajjak@gmail.com (R.R.S.); yazdanpanah.mohammad1991@gmail.com (M.Y.); 2 Swedish University of Agricultural Sciences, P.O. Box 7070, SE-750 07 Uppsala, Sweden; isak.rajjak@gmail.com; 3 Department of Chemistry, College of Science, King Saud University, P.O. Box 2455, Riyadh 11451, Saudi Arabia; krais@ksu.edu.sa; 4 High-Performance Computing Unit (CECC-HCU), Center of Excellence on Catalysis and Catalytic Reaction Engineering (CECC), Department of Chemical Engineering, Faculty of Engineering, Chulalongkorn University, Bangkok 10330, Thailand; supareak.p@chula.ac.th

**Keywords:** CO_2_ conversion, polymers, liquid phase, catalyst deactivation, green chemistry

## Abstract

Benzoxazine-based polymer (PBZ) acts as a catalyst for converting CO_2_ into cyclic carbonates. PBZ-ODA was successfully synthesized and examined for its catalytic efficiency, proving to be effective under milder conditions with higher yields at room temperature without solvents. Various terminal monoepoxides showed good to excellent conversion rates, while epoxides with aromatic or bulky groups and second oxirane rings also were able to produce corresponding cyclic carbonates. Recyclability tests demonstrated that regenerated PBZ-ODA retained 93% of its catalytic activity. Overall, there was a low catalyst deactivation, as investigated by chemical experiments, SEM-EDX, TGA, FT-IR, XPS, XRD and NMR. The catalyst is reusable and suitable for use in flow or batch reactors.

## 1. Introduction

Carbon dioxide (CO_2_) emission is a significant concern for our planet since it contributes negatively to global climate change. The chemical fixation of CO_2_ is an important solution to the problems of rising energy needs and global warming. In general, CO_2_ capture, storage, and usage are critical to our long-term sustainability [1,2,3,4,5,6]. CO_2_, which comes from combustion waste, can be used as a carbon source to make chemicals and fuels, making it a key area of study [7]. CO_2_ is very stable, and a significant amount of energy is needed to activate and change it into other organic molecules. The energy needed to break the C=O bond in CO_2_ is 750 kJ mol^−1^, which is higher than other bonds like C−H and C−C. Therefore, the effectiveness of CO_2_ conversion processes largely relies on the type of catalyst used. The chemical transformation of CO_2_ into useful products is of high interest and also, feasible due to its easy availability as a C1 resource [8,9,10,11]. The 100% atom-economical cycloaddition of CO_2_ with epoxides to produce cyclic carbonate stands out as one of the most promising and greener ways of CO_2_ utilization that has several applications [12]. These products are used in various areas like solvents, extracting agents, and high-energy batteries due to their stability, solubility, and low toxicity, aligning with green chemistry principles [13].

Several types of catalysts have been tested for the cycloaddition of CO_2_ with epoxides, including organocatalysts, MOFs, organometallic compounds, organic and inorganic polymers [14,15,16,17,18,19,20,21]. Many of these transformations require nucleophilic counterions, including commonly used tetrabutylammonium bromide (TBAB) and tetrabutylammonium iodide (TBAI). Cost-effective polymerization methods create crosslinked polymers with large surface areas for CO_2_ conversion [22,23,24]. Moreover, most of these catalytic processes demand harsh conditions. The requirement of high reaction temperature, high CO_2_ pressure and the use of expensive catalysts limits the potential use of these efficient catalytic methods at the industry level. Additionally, the use of harsh conditions leads to catalyst leaching and deactivation, and thus, it blocks the possibility of the recyclability of catalysts. It is of paramount importance that new catalytic processes must be developed in a way that is green and economically feasible. Homogeneous catalysts show high yield but have issues with separation and recycling [25]. Organic polymers can be easily synthesized, and their physical and chemical properties can be conveniently tuned by changing the functionalities of the monomer. Consequently, such polymers stand as a viable option to be used as heterogeneous catalysts in CO_2_ conversion.

In this work, we have prepared a polybenzoxazine-based catalyst (PBZ-ODA) containing hydroxy functionality and tested its catalytic performance in combination with quaternary ammonium salts as cocatalysts at room temperature and atmospheric CO_2_ pressure. PBZ-ODA exhibited promising catalytic activity in terms of yield, selectivity, recyclability and stability. This transformation using PBZ-ODA catalyst was found to be effective with a variety of epoxides, such as aliphatic, diepoxide and epoxides with aromatic and unsaturated substituents at RT with no requirement of reaction solvent.

## 2. Results and Discussion

**Scheme 1:**
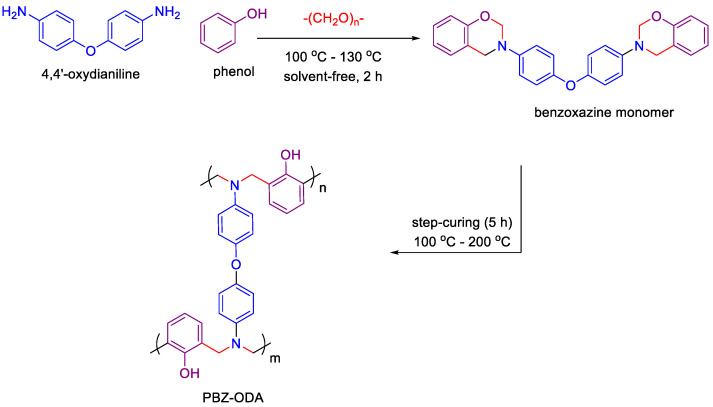
Synthetic steps for PBZ-ODA.

The polybenzoxazine, PBZ-ODA, was synthesized following the steps shown in Scheme 1. A solvent-free multicomponent one-pot reaction of phenol, paraformaldehyde and 4,4′-oxydianiline resulted in the formation of benzoxazine monomer. A heat-induced ring-opening polymerization reaction leads to the desired polymer, PBZ-ODA.

**Figure 1:**
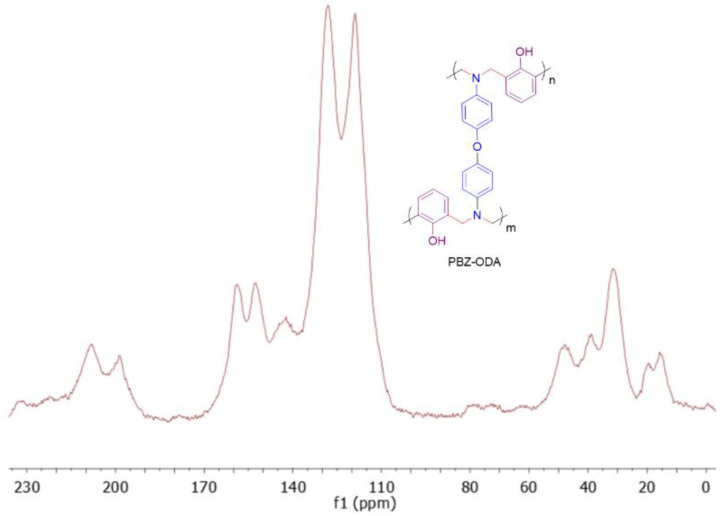
^13^C-NMR of PBZ-ODA.

The ^13^C-NMR shows the aromatic peaks in the range of δ 110–220 ppm and peaks for aliphatic benzylic carbons of the polymer can be seen in the range of δ 10–60 ppm (Figure 1) [26,27].

**Figure 2:**
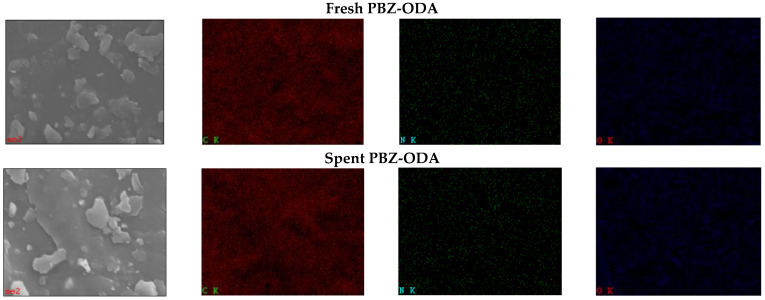
SEM images with elemental mapping of fresh and spent PBZ-ODA.

The morphology and elemental mapping of the fresh and spent PBZ-ODA were carried out by scanning electron microscopy (SEM), and the results are presented in Figure 2. Elemental mapping images show uniform dispersion of C, N, and O in PBZ-ODA, indicating an even distribution of phenolic and oxydianiline units in the polymer structure. Interestingly, the distribution and composition of C, N and O were similar in fresh and spent PBZ-ODA (Figure 2 and Table 1). The weight ratio of C/O and C/N was similar for both fresh and spent catalysts with below 4% difference.

**Figure 3:**
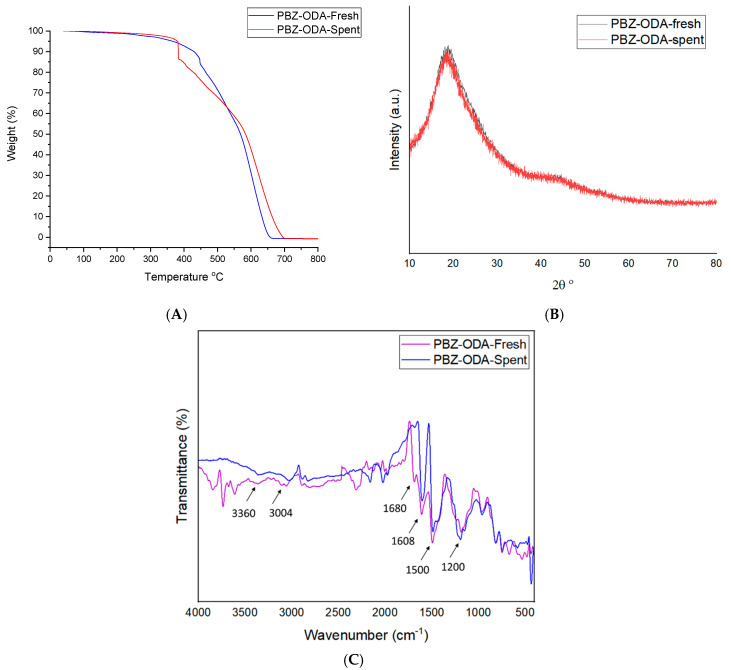
TGA (**A**), XRD (**B**) and FT-IR (**C**) for both fresh and spent PBZ-ODA.

The thermogravimetric analysis (TGA) of PBZ-ODA displayed excellent thermal stability of PBZ-ODA with a thermal degradation in the range of 390—460 °C for both fresh and spent PBZ-ODA (Figure 3A). Moreover, as seen from the TGA graph, both fresh and spent catalysts showed no weight loss whatsoever up to 200 °C. At above 200 °C, very minute changes in weight loss were observed for both fresh and spent catalysts. Furthermore, a sudden weight loss was observed for the spent catalyst at approximately 390 °C, which can be attributed to unavoidable structural changes or diffusion of reactants/impurities from the previous test run [28]. In the case of fresh PBZ-ODA, a very low decrease was observed until 460 °C. This indicates that the polymeric catalyst is thermally stable and can be used for high-temperature reactions. XRD of the catalyst shows a broad peak at 2θ = 18.5° for pure PBZ, indicating its amorphous nature, widening from 12° to 34°. Moreover, no significant change was observed for both fresh and spent catalysts (Figure 3B). The FTIR spectra of fresh and spent catalysts provide additional evidence of polybenzoxazine formation and stability (Figure 3C). The C=C stretching vibrations are observed at ~1608 cm^−1^. The tri-substituted benzene ring vibration bands for benzoxazine appear at ~1500 cm^−1^. The C–O vibration band at ~1200 cm^−1^ confirms the formation of the polyether structure post-synthesis in fresh and its preservation in spent catalysts. Additionally, stretching vibrations of aromatic C–H (~3004–3062 cm^−1^) and aromatic C=C (~1492–1680 cm^−1^) are detected in both fresh and spent catalysts. The peak at ~3350–3360 cm^−1^ indicates the asymmetric stretching -OH in both fresh and spent catalysts [29,30].

**Figure 4:**
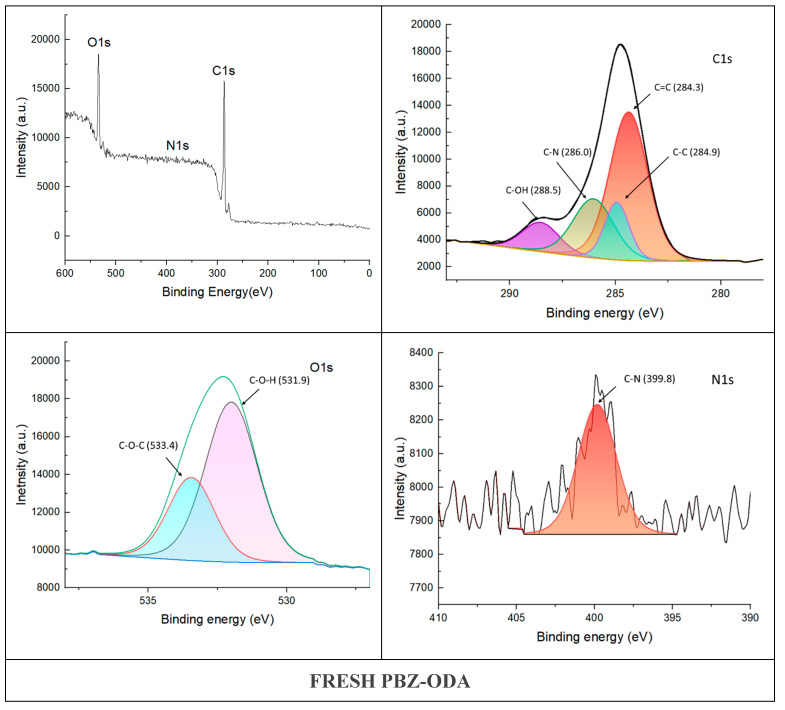

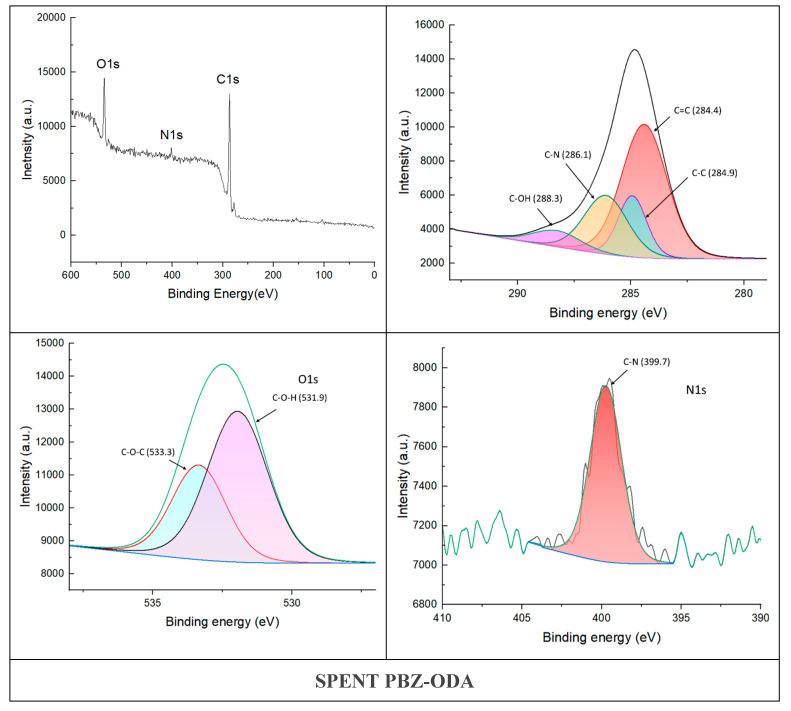
XPS spectra of fresh and spent PBZ-ODA with core-level spectra of C1s, N1s and O1s.

XPS analysis was performed for further characterization of both fresh and spent PBZ-ODA catalysts (Figure 4) and was compared with previously reported data [31,32,33]. Wide-scan XPS showed signals of C, O and N, indicating the presence of PBZ chains. The C_1s_ spectrum revealed specific peaks for C=C, C-C, C-N and C-OH at 284.3, 284.9, 286 and 288.5 eV for fresh PBZ-ODA and 284.4, 284.9, 286.1 and 288.3 eV for spent PBZ-ODA, respectively, aligning with PBZ structure. The O_1s_ spectrum displayed specific peaks for C-O-H and C-O-C at 531.9 and 533.4 eV for fresh PBZ-ODA and 531.9 and 533.3 eV for spent PBZ-ODA, respectively. Furthermore, N_1s_ spectrum showed a specific peak for C-N at 399.8 eV for fresh PBZ-ODA and 399.7 eV for spent PBZ-ODA. Overall, the results support the formation of PBZ chains.

**Scheme 2:**
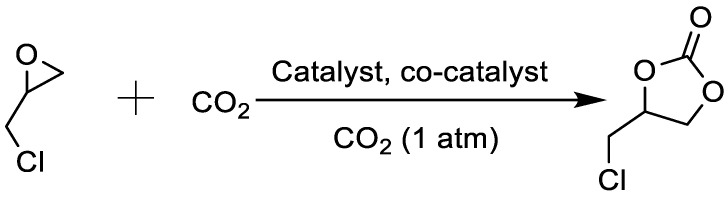
Cycloaddition of CO_2_ and epichlorohydrin (ECH) to produce epichlorohydrin carbonate (EC).

**Figure 5:**
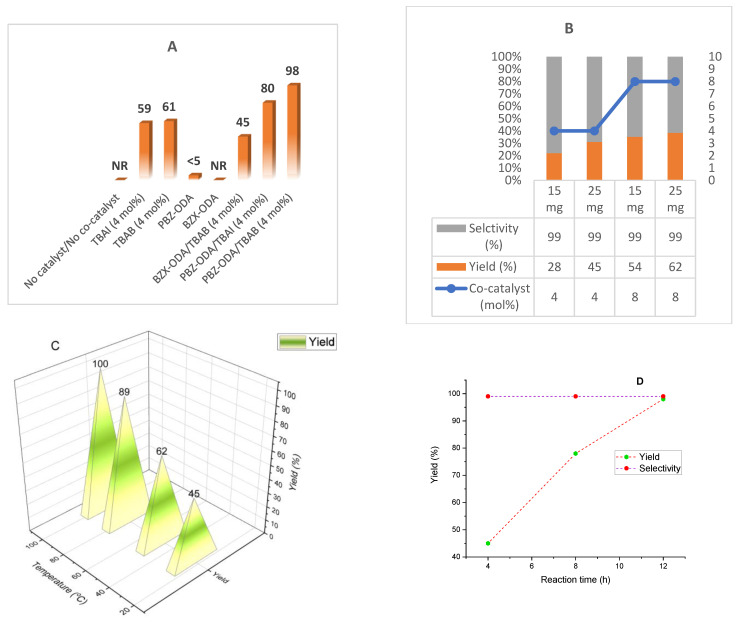
Optimization studies for PBZ-ODA-catalyzed cycloaddition of CO_2_ and epichlorohydrin: (**A**) tests to find high-yielding catalyst/catalytic system at RT for 12 h; (**B**) catalyst dosage study at RT for 4 h using TBAB as cocatalyst; (**C**) effect of reaction temperature for reaction run of 4 h and (**D**) reaction progress with time at RT.

The synthesized heterogeneous polymeric catalyst, PBZ-ODA, was used to convert CO_2_ into cyclic carbonates via the cycloaddition of CO_2_ with epoxides under mild reaction conditions. Typically, 5 mmol of epichlorohydrin, 25 mg of PBZ-ODA and 4 mol% of TBAI or TBAB were taken into a round-bottom flask with a CO_2_ balloon (Scheme 2). The cycloaddition reaction was allowed to proceed for 12 h at room temperature without any solvent. Substrate conversion was analyzed by NMR of the crude product. The outcomes are summarized in Figure 5 and Supplementary SI-1.

As expected, no conversion was observed in the absence of both PBZ-ODA and cocatalyst and using the monomer alone as a catalyst (Figure 5A). Using only nucleophilic cocatalysts, TBAI and TBAB, the epichlorohydrin conversion was found to be 59% and 61%, respectively. Almost no conversion was noted in the presence of PBZ-ODA alone, indicating no effect of the nucleophilicity of tertiary amine present in PBZ-ODA. As compared to individual use of TBAI and TBAB, when combined with monomer, the conversion of ECH was decreased. This suggests that monomers led to a decrease in the catalytic activity of cocatalysts. Interestingly, PBZ-ODA/TBAI combination led to an increase in conversion (80%), and PBZ-ODA/TBAB gave an almost quantitative conversion of ECH to EC (98%).

The CO_2_ cycloaddition reaction is affected by catalyst amount, temperature, time and pressure. PBZ-ODA/TBAB with the highest conversion was used to study how reaction conditions affect catalytic performance, as shown in Figure 5B–D and ^1^H-NMR spectra are included in Supplementary SI-2, SI-3 and SI-4, where EC achieved above 99% selectivity consistently in all conditions. For optimization studies, reaction time was fixed to be 4 h to gain a clear distinction between various parameters that are being implemented. As shown in Figure 5B, EC yields increased from 28% to 45% with catalyst dosage increases from 15 mg to 25 mg while using 4 mol% of TBAB. Moreover, an additional cocatalyst enhances PBZ-ODA activity towards cycloaddition. The yields of EC rose from 28% to 54% when using PBZ-ODA 15 mg and 8 mol% of TBAB, while EC yield increased from 45% to 62% when using PBZ-ODA 25 mg and 8 mol% of TBAB. The ECH conversion was studied with respect to the temperature and reaction time (Figure 5C,D). An increase in temperature was directly proportional to ECH conversion. The ECH conversion was found to be 62%, 89% and 100% at reaction temperatures of 50 °C, 80 °C and 100 °C, respectively (Figure 5C). Similarly, the EC yields rose as reaction time increased, peaking at 98% within 12 h (Figure 5D). Optimal CO_2_ cycloaddition conditions include 25 mg PBZ-ODA, 4 mol% TBAB, 1 bar CO_2_, and 12 h, achieving 98% yield and over 99% EC selectivity. The EC was isolated using silica-column chromatography with a hexane/ethyl acetate mixture, achieving an isolated yield of 91%.

We tested the substrate scope of our catalytic approach. Various epoxides gave satisfactory to excellent results using PBZ-ODA/TBAB as a bifunctional catalytic system. The obtained results are summarized in Table 2. As can be seen, there was a noticeable effect on the conversion when the nucleophilic halogen of the cocatalyst changed from iodine (TBAI) to bromine (TBAB). When aliphatic epoxides, epichlorohydrin, propylene oxide and glycidol, were subjected to this cycloaddition reaction, the results were promising with yields of corresponding cyclic carbonates of 98%, 76% and 91%, respectively (Table 2, entry 1–3). Propylene carbonate and glycidol carbonate have major markets for producing commercially useful polycarbonates and polyethers. Lower conversion was observed for hexene oxide (30%) and when phenyl-protected glycidol was subjected to this cycloaddition (29%) (Table 2, entries 4 and 6). Styrene oxide also gave a lower conversion of styrene carbonate (12%) (Table 2, entry 7). Interestingly, 1,7-octadiene diepoxide, which can give monocyclic or bicyclic carbonate, was converted only into a corresponding bicyclic carbonate (Table 2, entry 8). Furthermore, 1,3-butadiene monoepoxide resulted in the production of a corresponding cyclic carbonate having an alkene moiety—an interesting molecule with applications in polymer chemistry to produce polymers with pendant cyclic carbonate rings that can further undergo functional group transformation to produce a library of different polymers (Table 2, entry 5). The PBZ-ODA/TBAB system was found to be limited when employed for internal epoxides such as epoxycyclohexane and cyclopentene oxide and gave only traces of products (Table 2, entries 9 and 10). The lower catalytic efficiency of PBZ-ODA for internal epoxides compared to terminal epoxides can be attributed to the bulkiness and steric hindrance of the substituents, which restricts the nucleophilic attack of the epoxide ring. Moreover, it can also be duly noted that, in this work, the catalytic process is tested under very mild reaction conditions. Further improvements can be achieved by testing with relatively higher but mild reaction temperatures or CO_2_ pressures to obtain higher conversion. Since the PBZ-ODA is thermally stable and has no detrimental effect on its chemical composition, as seen from SEM-EDX and TGA analysis, the catalyst can be used at high reaction temperatures and under high-pressure conditions for a prolonged time.

**Chart 1:**
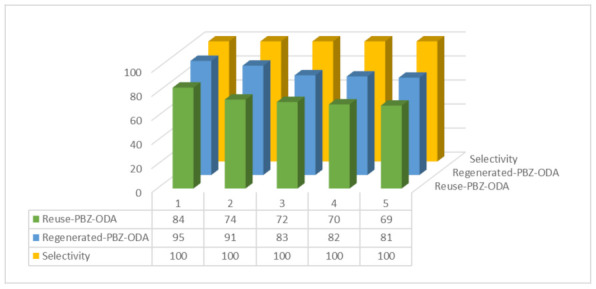
Recyclability tests of PBZ-ODA/TBAB for CO_2_ cycloaddition to ECH. Reaction conditions: epoxide (5 mmol), PBZ-ODA (25 mg), TBAB (4 mol%), CO_2_ (1 atm.), RT, 12 h.

**Figure 6:**
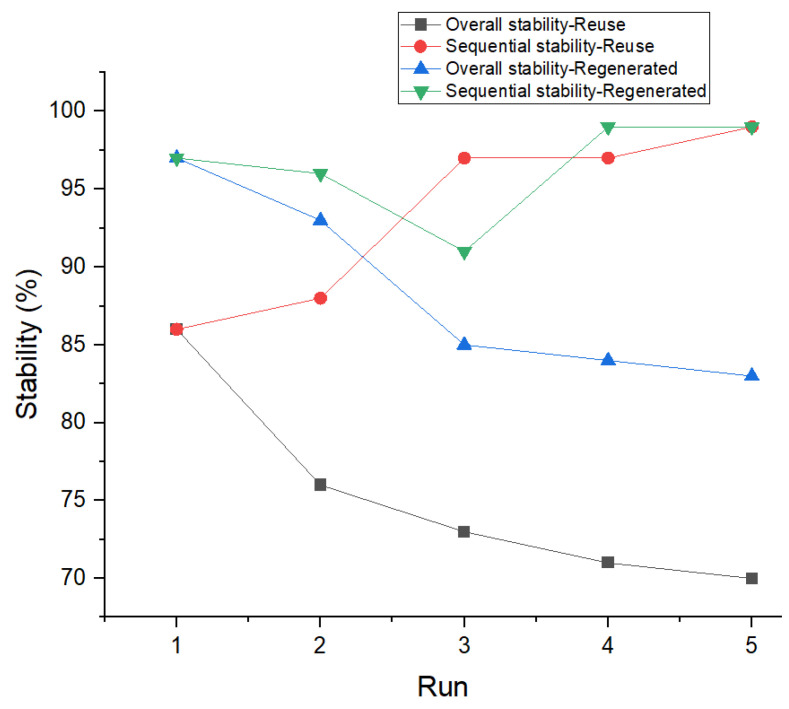
Catalyst stability was evaluated based on cyclic carbonate production compared to fresh catalyst (black and blue lines, indicating overall stability) and catalyst from the previous test run (red and green lines, indicating stability by comparing the yield of two consecutive reactions).

The industrial viability of heterogeneous catalysts depends on their stability and recyclability. The catalyst PBZ-ODA was tested for its reusability and results are shown in Chart 1. The catalyst was filtered after the reaction and reused for five consecutive runs (represented as Reuse-PBZ-ODA in Chart 1 and Figure 6). Furthermore, we studied the effect of regeneration of the used catalyst (represented by Regenerated-PBZ-ODA in Chart 1 and Figure 6). The catalyst was regenerated after each reuse by subjecting it to oven-drying, following the same procedure as during the synthesis of PBZ-ODA (drying in a ventilated oven; 100 °C for 1 h, 120 °C for 1 h, 140 °C for 1 h, 160 °C for 1 h and 200 °C for 1 h).

Results showed that PBZ-ODA worked effectively for up to five reuses (Chart 1). The first reuse had a decrease in catalytic activity of 18%, with 84% conversion compared to 98% with a fresh catalyst. The next four reuses yielded 74%, 72%, 70% and 69%, highlighting comparable catalytic activity. In contrast, regenerated PBZ-ODA had better results, losing only 7% yield in its first use and achieving 95% conversion. The last three runs maintained the catalytic performance, giving 83%, 82% and 81% conversion.

Recyclability studies show that PBZ-ODA is a stable and effective catalyst. During its first reuse, the activity of PBZ-ODA dropped, producing 14% less EC than the fresh catalyst. However, in the next four reuses, the yield loss was less than 5%, and catalytic activity remained consistent (Figure 6, red line). Some optimization is necessary since the fifth reuse saw a 30% decrease in production compared to the fresh catalyst (Figure 6, black line). Regenerated PBZ-ODA initially produced only 7% less EC than a fresh catalyst, with the last three uses showing less than 2% loss in yield (Figure 6, blue and green lines). Overall, the results indicate the stability of PBZ-ODA and its potential for industrial use.

To enhance performance, the catalysts must be studied in-depth, as there was a 32% decrease in product generation in its fifth reuse of PBZ-ODA and 21% for the fifth reuse of PBZ-ODA-Spent when compared with fresh PBZ-ODA (Figure 6, red and blue line, respectively). The catalytic deactivation can mainly originate from the unavoidable loss and decomposition of the catalyst during the cycling process. Furthermore, the SEM-EDX and TGA results for fresh and spent catalysts did not change significantly. Additionally, no catalyst was decomposed or leached during the reaction, as no traces or presence of benzoxazine peaks in ^1^H-NMR during recyclability studies were observed (Supplementary SI-5 and SI-6). The study found that the catalyst PBZ-ODA maintained its composition and properties, indicating strong cyclic stability.

**Scheme 3:**
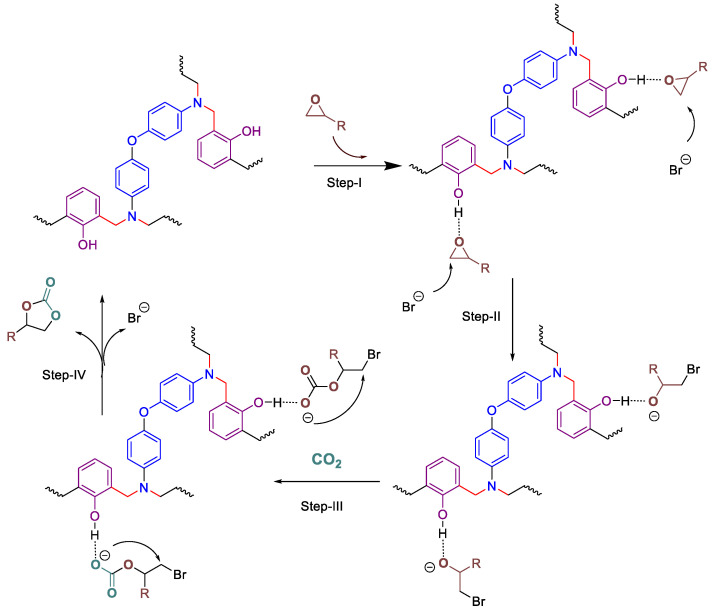
Proposed mechanism for PBZ-ODA/TBAB catalyzed cycloaddition of CO_2_ and epoxide.

As shown in Scheme 3, the catalytic mechanism of polybenzoxazine involves the adsorption and activation of multiple epoxide molecules through the interaction between phenolic protons and oxygen atoms (Step-I) [34]. This forms a ring-opening intermediate through nucleophilic bromide attack on the less hindered β-carbon, stabilized by acidic phenol hydrogen (Step-II). CO_2_ reacts with the intermediate to generate acyclic esters (Step-III), which then undergo intramolecular cyclization to produce cyclic carbonates and release bromide (Step-IV). The catalyst then starts the next reaction cycle.

In comparison to previous polymer-based catalyst studies, the PBZ-ODA/TBAB catalyst shows superior catalytic activity for the CO_2_ and epoxides cycloaddition reaction. Table 3 summarizes the results, indicating that PBZ-ODA requires lower catalyst loading, reaction temperature and CO_2_ pressure, with shorter reaction times for some of these reported research works. Most of these selected catalytic processes required atmospheric CO_2_ pressure, similar to this work. However, as can be seen, the elevated reaction temperature was needed to achieve EC with high yield (Table 3, entries 1, 2, 4 and 5). For some of these catalytic processes, the reaction time was shorter or the same to achieve considerably good conversion, but a higher reaction temperature was needed (Table 3, entries 1, 2 and 4). Interestingly, PBZ-ODA achieves a high 98% ECH conversion rate and produces cyclic carbonate with excellent selectivity. These findings highlight the efficiency of PBZ-ODA as a catalyst for the synthesis of valuable products. This catalytic system can be further improved by developing or modifying it into a single-component catalytic system without the need for any cocatalyst, as in the case of entries 1, 2 and 5 (Table 3).

## 3. Materials and Methods

### 3.1. Materials

Chemicals from Sigma-Aldrich (St. Louis, MO, USA) and TCI (Tokyo, Japan) were used as purchased without purification.

### 3.2. Catalyst Preparation

The benzoxazine monomer was synthesized using the solventless procedure by mixing the phenol, 4,4′-oxydianiline (ODA), and paraformaldehyde in 2:1:4 molar ratio. The mixture was heated on a hot plate firstly at 100 °C for 1 h and later at 130 °C for 1 h with constant magnetic stirring, and the dark yellow viscous mixture obtained was allowed to cool down at room temperature to obtain a brittle dark yellow benzoxazine monomer. The polybenzoxazine, PBZ-ODA, was obtained by curing the obtained monomer using the following step-curing in a ventilated oven; 100 °C for 1 h, 120 °C for 1 h, 140 °C for 1 h, 160 °C for 1 h, and 200 °C for 1 h.

### 3.3. Cycloaddition Reaction of CO_2_ with Epoxides

In a 50 mL round-bottom Schlenk flask equipped with a magnetic stirrer, the epoxide substrate (5 mmol) was placed. The nucleophilic cocatalyst (4 mol%) and PBZ-ODA (25 mg) were added. A rubber balloon of CO_2_ was attached to the Schlenk flask, and some of the CO_2_ was used to flush the flask to replace air. The reaction vessel was tightly sealed to avoid any CO_2_ leakage and stirred at room temperature for 12 h. Following this period, a sample of the reaction mixture was added to an NMR tube after removing PBZ-ODA via filtration to evaluate the substrate conversion using ^1^H-NMR spectroscopy in CDCl_3_.

The morphological properties of catalysts were obtained on a scanning electron microscopy (SEM) equipped with an energy dispersive X-ray (EDX) to observe the structure and to analyze the elemental distribution.

The thermogravimetric analysis (TGA) was performed to understand the thermal stability of the catalyst. Nuclear Magnetic Resonance (NMR) spectroscopy was used for ^1^H-NMR and ^13^C-NMR analysis.

## 4. Conclusions

We have synthesized the phenolic catalyst PBZ-ODA via ring-opening polymerization for epoxide activation, achieving the desired functionality. The combined catalytic system, PBZ-ODA/TBAB, showed promising catalytic performance in the cycloaddition of CO_2_ with various epoxides under mild reaction conditions (RT, 1 bar CO_2_, 12 h), achieving up to 98% yield and >99% selectivity. The catalyst PBZ-ODA shows excellent substrate compatibility and recyclability in substrate scope and reuse tests. Its superior catalytic activity was confirmed through comparisons with other polymeric catalysts, making it a promising option for converting CO_2_ into cyclic carbonates efficiently. Future research will focus on optimizing the catalyst to enhance performance, particularly addressing limitations in cycloaddition reactions and exploring the use of flue gas as a CO_2_ source. These improvements can be achieved by turning the functionality of PBZs by changing the type of amine and phenol derivatives used in the synthesis of monomers. Such processes would lead to green industrial processes to achieve efficiently value-added products.

## Figures and Tables

**Table 1:** Elemental distribution/composition of fresh and spent PBZ-ODA.

	Fresh PBZ-ODA		Spent PBZ-ODA	
*Element*	*Wt*%	*At*%	*Wt*%	*At*%
***C***	68.01	73.21	68.69	73.81
***N***	08.21	07.58	08.12	07.48
***O***	23.78	19.22	23.19	18.71

**Table 2:** Substrate scope for the cycloaddition of CO_2_ and epoxide.

Sr. No.	Epoxide	Cyclic Carbonate	Yield (%) *
1	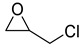	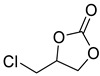	98%
2	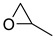	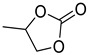	76%
3	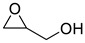	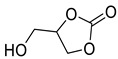	91%
4	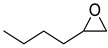	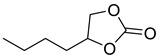	30%
5	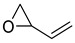	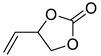	89%
6	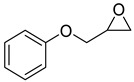	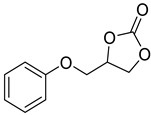	29%
7	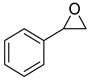	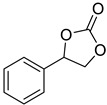	12%
8	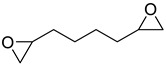	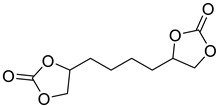	21%
9	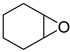	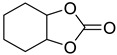	traces
10	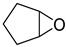	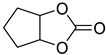	traces

* based on ^1^H-NMR.

**Table 3:** Comparative data of the cycloaddition of CO_2_ and ECH.

Sr. No.	Catalyst/Cocatalyst Loading	CO_2_ Pressure (bar), Temperature (°C), Time (h)	ECH Conversion (%)	Ref.
1	[HBIM-6]Br-DCX(3) (5.54 mol%)	1 140 1	99 *	[34]
2	poly(HCO_3_-OH-2) (0.15 g)	1 80 12	90 **	[35]
3	MOP-0 0.05 g/KI or TBAI (5 mol%)	10 100 24	89 **	[36]
4	CETB-C_16_Br (0.025 g)/TBAB (0.75 mol%)/HBr (1 mol%)	1 80 6	93.2 **	[26]
5	Pyridyl salicylimine-based polymeric organocatalyst (0.03 g)	1 100 24	99 *	[27]
6	PBZ-ODA (0.025 g)/TBAB (4 mol%)	1 RT (ca. 25 °C) 12	98 *	This work

* based on ^1^H-NMR; ** based on GC.

## Data Availability

The original contributions presented in this study are included in the article/Supplementary Materials. Further inquiries can be directed to the corresponding author(s).

## Supplementary Materials

The supporting information can be downloaded at: https://www.mdpi.com/article/10.3390/ijms26031111/s1.
