# Supplementary material for: Catalytic Performance of Oxydianiline-Derived Polybenzoxazine in the Cycloaddition of CO2 with Epoxides for Selective and Cleaner Production of Cyclic Carbonates
Source: Int J Mol Sci. 2025 Jan 27;26(3):1111. doi: 10.3390/ijms26031111 (PMC11817366; doi:10.3390/ijms26031111)

# Catalytic performance of oxydianiline-derived polybenzoxazine in the cycloaddition of $CO_2$ with epoxides for selective and cleaner production of cyclic carbonates

**Calculation of conversion and selectivity**:

$^1$H NMR methods for calculation of conversion and selectivity:

In the reaction below, the blue arrows indicate the peaks belonging to the epoxide and the red arrows point toward the cyclic carbonate products peaks. The conversion of epoxide was determined by comparison of the $^1$H NMR integrals of the starting material ($H_a$) and epichlorohydrin carbonate (EC) protons ($H_b$).

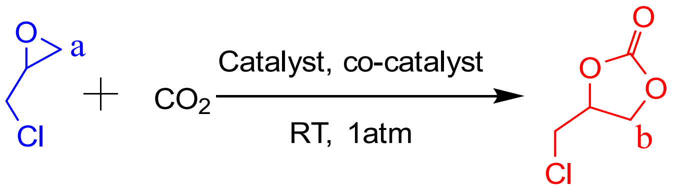


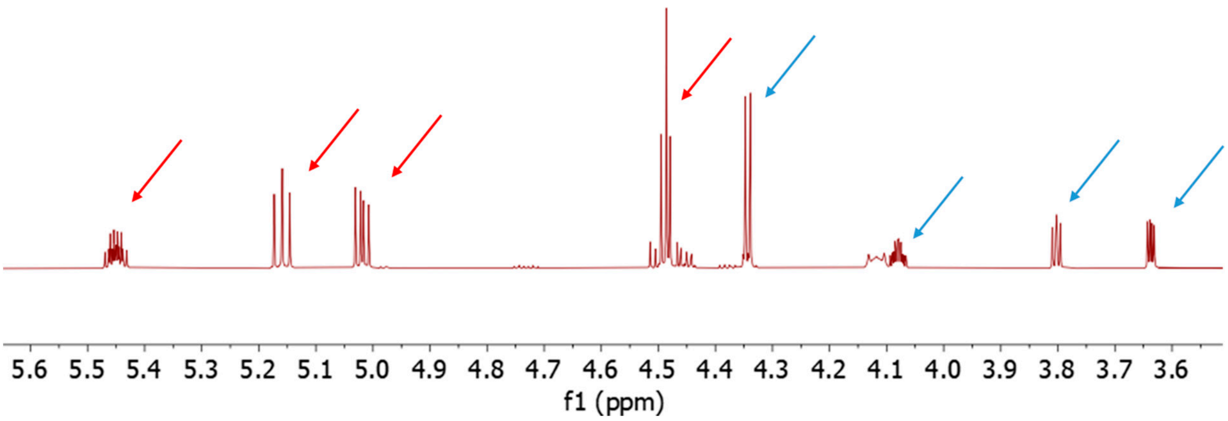

*SI- S1: Optimization studies for finding the catalyst/catalytic system**

| **Catalytic system** | **$^1$H-NMR spectra** |
| --- | --- |
| PBZ-ODA (25 mg)/TBAB (4 mol%) | 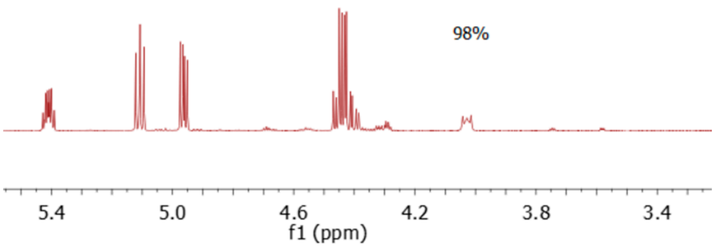 |
| PBZ-ODA (25 mg)/TBAI (4 mol%) | 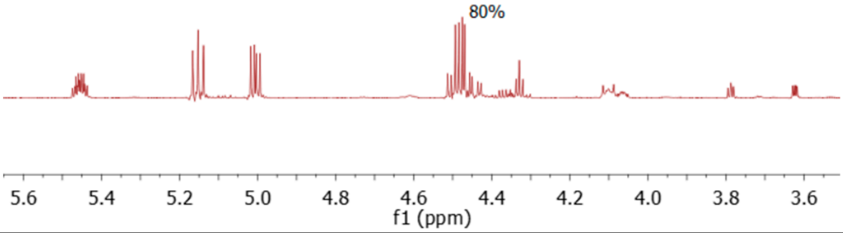 |
| BZX monomer (25 mg)/TBAB (4 mol%) | 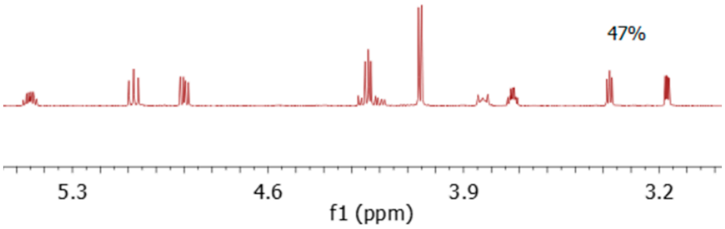 |
| TBAI (4 mol%) | 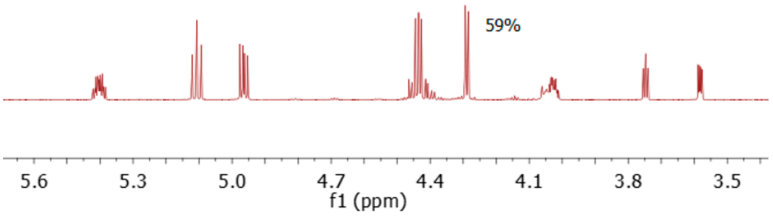 |
| TBAB (4 mol%) | 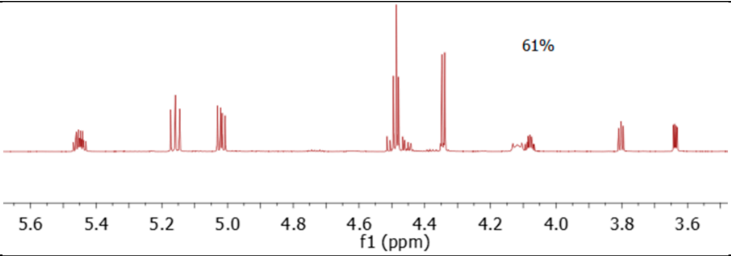 |

***Reaction conditions:** catalyst, co-catalyst (4 mol%), RT, 12 h, 1 bar $CO_2$

***SI- S2: Effect of catalyst/co-catalyst dosage on ECH conversion****

| Catalyst dosage | $^{1}$H-NMR spectra |
|---|---|
| PBZ-ODA (15 mg)/TBAB (4 mol%) | 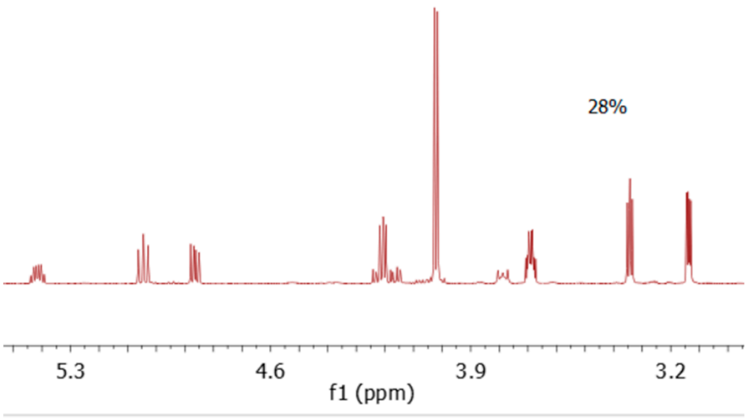 |
| PBZ-ODA (25 mg)/TBAB (4 mol%) | 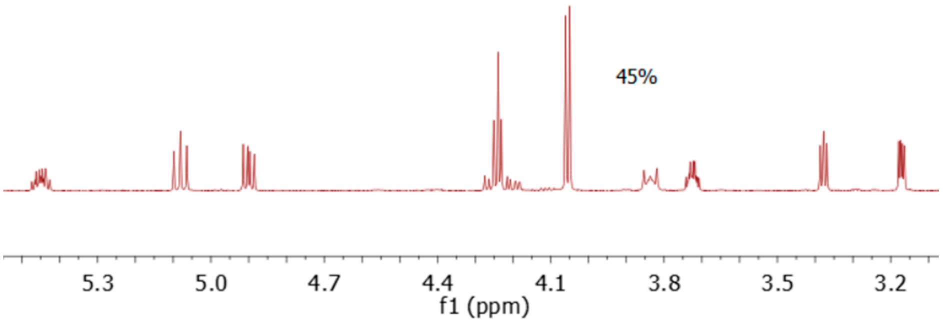 |
| PBZ-ODA (15 mg)/TBAB (8 mol%) | 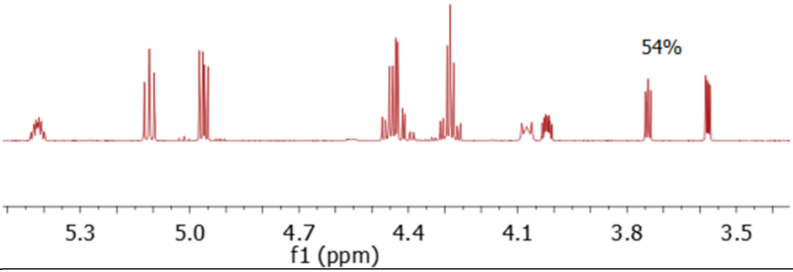 |
| PBZ-ODA (25 mg)/TBAB (8 mol%) | 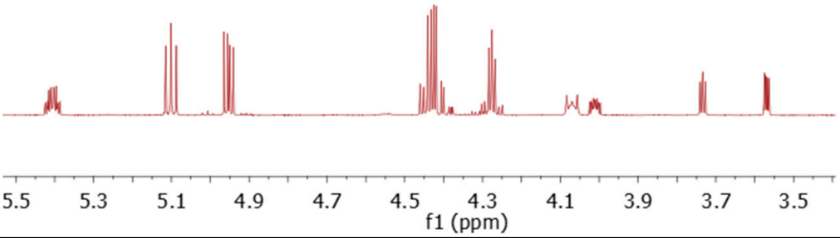 |

***Reaction conditions:** PBZ-ODA/TBAB, RT, 4 h, 1 bar $CO_2$

*SI- S3: Effect of reaction temperature on ECH conversion**

| **Reaction temperature** | **$^1$H-NMR spectra** |
|---|---|
| RT (ca. 25 ºC) | 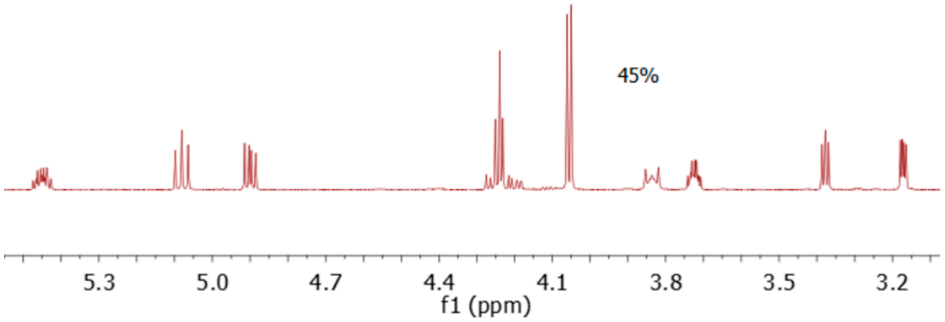  |
| 50 ºC | 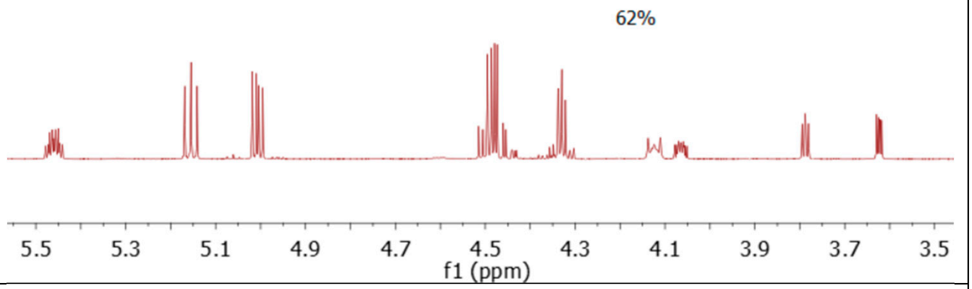  |
| 80 ºC | 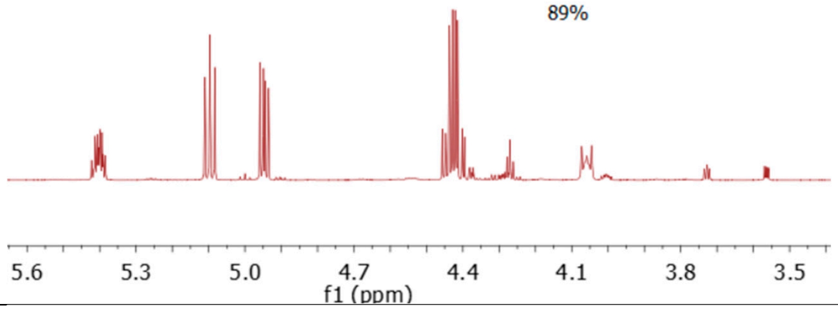  |
| 100 ºC | 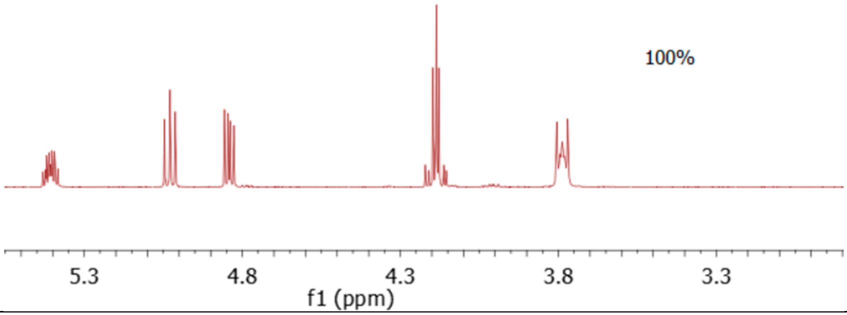  |

***Reaction conditions:** PBZ-ODA (25 mg)/TBAB (4 mol%), 4 h, 1 bar $CO_2$

*SI- S4: Reaction progress with time**

| Reaction time | $^{1}H$-NMR spectra |
|---|---|
| 4 h | 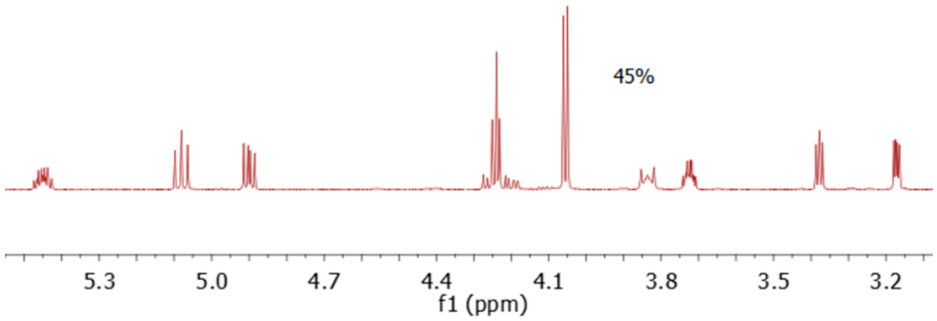  |
| 8 h | 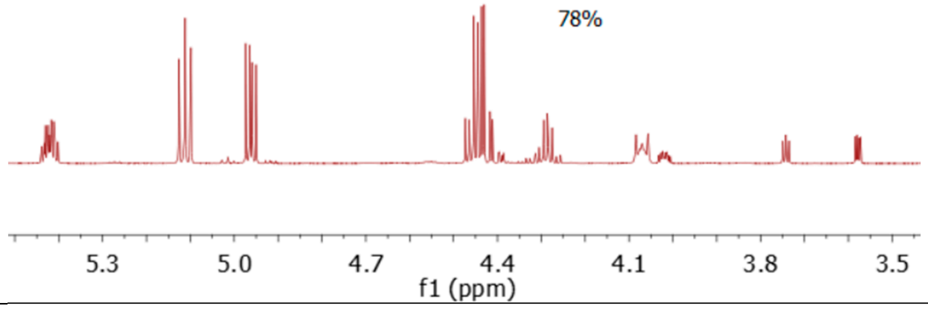  |
| 12 h | 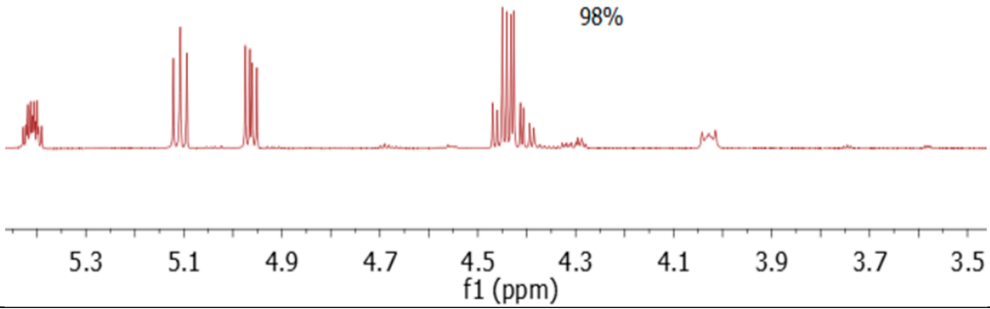  |

***Reaction conditions:** PBZ-ODA (25 mg)/TBAB (4 mol%), RT, 1 bar $CO_2$

*SI- S5: Reusability of PBZ-ODA*

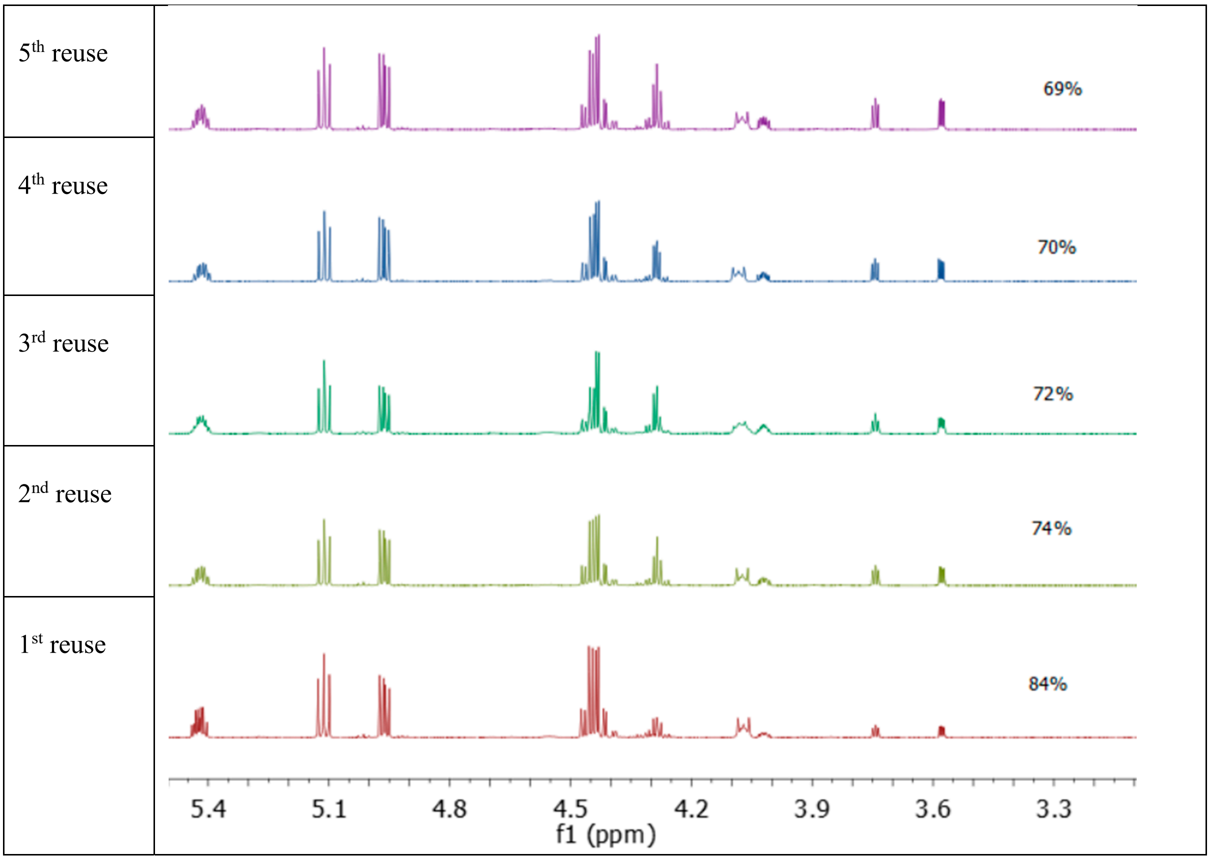


*SI- S6: Reusability of regenerated PBZ-ODA*

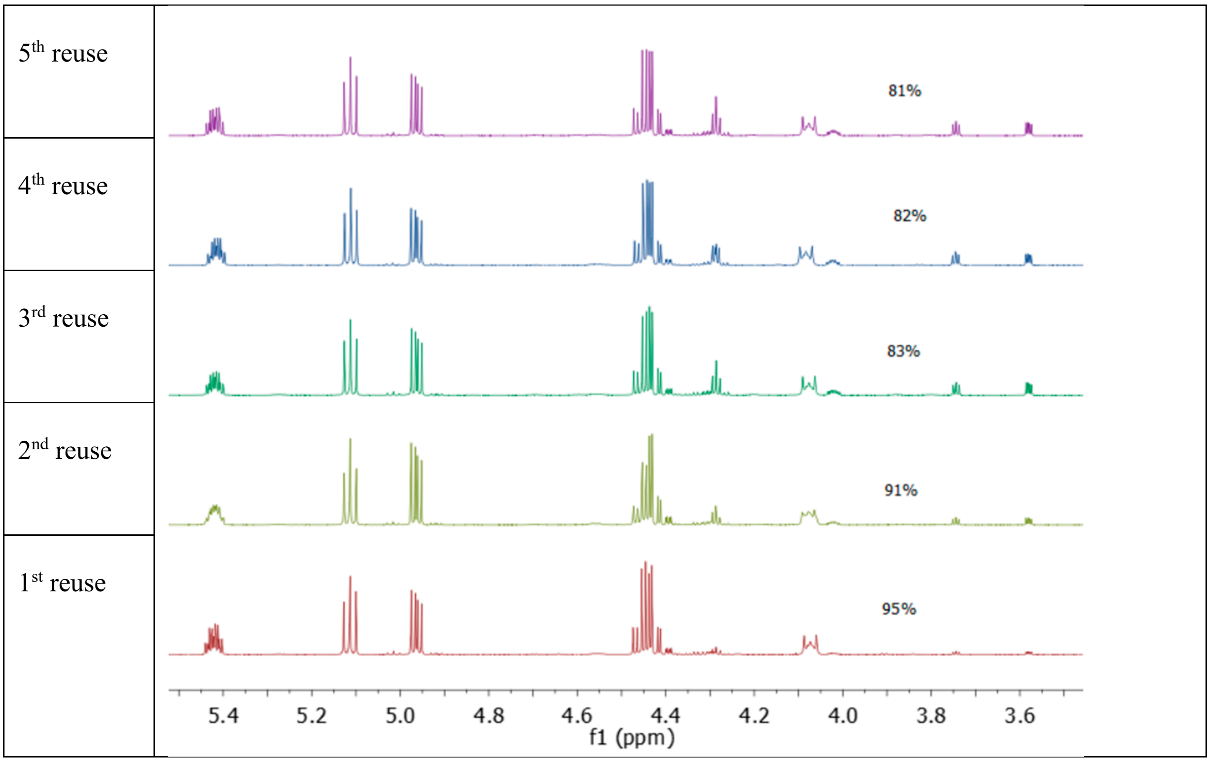

Supplement: Supplementary file 1 [file ijms-26-01111-s001.zip › ijms-3407011-supplementary.pdf]
